# Improved Diagnostic Confidence Imparted by Radiologists in Radiology Reports After Educational Interventions on Reporting Styles

**DOI:** 10.7759/cureus.53926

**Published:** 2024-02-09

**Authors:** Uffan Zafar, Muhammad Nadeem Ahmad, Naila Nadeem, Mallick Muhammad Zohaib Uddin, Noman Khan, Muhammad Masood Alam, Anam Hafeez, Hafsa Pervez, Fariha Zafar

**Affiliations:** 1 Radiology, Aga Khan University Hospital, Karachi, PAK; 2 Medicine and Surgery, Dow University of Health Sciences, Civil Hospital Karachi, Karachi, PAK; 3 Epidemiology and Public Health, Quaid-E-Azam Medical College, Bahawalpur, PAK

**Keywords:** confidence, diagnostic certainty scale, certainty scale, dcps, diagnostic certainty phrases, reporting styles, radiology reports, hedging

## Abstract

Background

Radiology reports are important medico-legal documents facilitating communication between radiologists and referring doctors. Language clarity and precision are crucial for effective communication in these reports. Radiology reporting has changed with the evolution of imaging technology, prompting the adoption of precise terminology. Diagnostic certainty phrases (DCPs) play an important role in communicating diagnostic confidence in radiology reports.

Objective

The aim of this study was to evaluate the use of DCPs in radiology reports, before and after targeted educational interventions.

Materials and methods

The study was approved by the Aga Khan University Hospital's Ethical Review Committee and includes cross-sectional radiology reports. It involved three cycles of retrospective evaluation, with educational interventions in between to improve the use of DCPs.

Results

The study found a dynamic shift in the use of DCPs during the three cycles. Initially, intermediate-certainty phrases prevailed, followed by an increase in high-certainty phrases and a drop in low-certainty phrases. Later cycles showed a significant decline in DCPs and an increase in the use of definitive language. Across all subspecialties, there was a consistent decrease in intermediate- and low-certainty DCPs.

Conclusion

The study validates the transformative impact of educational interventions on the use of DCPs in radiology reports. The radiology reports frequently used DCPs with intermediate to low diagnostic certainty with improvement in the subsequent cycles of the study after educational interventions. It emphasizes the significance of continuing education to ensure the use of precise nomenclature.

## Introduction

A radiology report is not only an important medico-legal document but also a vital method of communication between radiologists and referring physicians [1,2]. Clarity of the language, as in other forms of communication, is essential for effective relaying of pertinent findings to the patient and referrer of the radiological request [3]. Radiology reporting has evolved over time, mirroring the advancements in imaging technology. There is now a heightened emphasis on the precise language within radiology reports. According to a Canadian survey of 200 clinicians, clarity and brevity were the most admired qualities of radiology reports [4].

“Hedging” refers to the use of unclear language or evasive statements in radiology reports to avoid definitive statements [1,5]. It is often a response to diagnostic uncertainty, challenges to interpretation in complex cases, fear of potential legal concerns, softening the impact of findings, or allowing room for the reinterpretation of evolving conditions. Hedging, often a balancing act between acknowledging uncertainty and providing useful information for patient care, can reduce the usefulness of radiology reports and should be kept to a minimum [2]. It is important for radiologists to appropriately convey the level of diagnostic certainty associated with the impression of the imaging report.

Radiology reports employ diagnostic certainty phrases (DCPs) to convey the degree of confidence. For example, the phrase “consistent with” conveys a greater degree of confidence than “suggestive of.” While these are intuitively understood, there may be wide variations in their perception between radiologists, primary care physicians, and patients [6-8]. As such, there have been attempts to standardize the lexicon and formulate a diagnostic certainty scale for improved and effective communication [9-11]. Attempts were also made to incorporate a diagnostic certainty scale into the reports, aiming to reduce inter-radiologist variation and enhance the clarity of diagnostic certainty to referring physicians and patients [12].

This study aimed to assess the impact of a targeted educational intervention on the use of DCPs and the diagnostic confidence imparted in radiology reports. It was hypothesized that such educational intervention could raise the radiologist’s awareness of appropriate use of DCPs, potentially leading to improved diagnostic confidence in radiology reports.

## Materials and methods

The Ethical Review Committee at Aga Khan University Hospital, Karachi, Pakistan, approved this study (Review Reference: 2023-8685-25456). All cross-sectional diagnostic radiology reports were included in the study. Diagnostic radiology reports with limitations such as significant artifacts, incomplete coverage of regions of interest, or lack of specific sequences were excluded from the study.

An educational intervention was designed focusing on the appropriate use of DCPs, adoption of a diagnostic certainty scale, and general recommendations on best radiology reporting practices. DCPs were categorized into high, intermediate, and low probabilities following the classifications outlined by Panicek and Hricak and by Shinagare et al. [13,14]. Phrases such as “consistent with,” “highly suggestive of,” “likely,” and “most likely” were grouped under high-probability DCPs. “Possible,” “possibly,” and “may represent” were classified as intermediate-probability DCPs. “May be,” “very unlikely,” and “unlikely” were categorized as low-probability DCPs. The study was performed in three cycles to determine the effectiveness of educational interventions.

Cycle 1

A sample of 40 radiology reports was selected using non-probability consecutive sampling in December 2021 before the educational intervention. The reports were analyzed for the number of overall DCPs, high-probability DCPs, intermediate-probability DCPs, and low-probability DCPs.

Cycle 2

An educational session was conducted by a senior resident, an instructor, and an attending radiologist in February 2022 for all residents, fellows, and attending radiologists. One month after the intervention, a sample of 50 radiology reports was collected using non-probability consecutive sampling. The reports were again analyzed for the number of overall DCPs, high-probability DCPs, intermediate-probability DCPs, and low-probability DCPs.

Cycle 3

A second short educational session was conducted in September 2023. One month after the second intervention, a sample of 50 cross-sectional radiology reports was collected. The reports were analyzed for the number of overall DCPs, high-probability DCPs, intermediate-probability DCPs, and low-probability DCPs.

The evaluation was undertaken by the same investigators in all three cycles, ensuring consistency in the assessment methodology. The investigators had at least two years of experience in radiology reporting. The combination of CT and MRI reports was chosen in each cycle to ensure a wide range of cases, covering a wide range of subspecialties and levels of complexity. While the number of reports was initially chosen without a formal power calculation, it was thought to be sufficient to discover important trends in DCP usage and provide preliminary insights into the effectiveness of educational interventions.

The data were analyzed in each cycle to assess changes in the usage of DCPs, with a particular emphasis on the usefulness of the educational interventions in boosting diagnostic confidence and precision in radiological reporting. Data analysis was conducted using IBM SPSS (Statistical Package for the Social Sciences) Statistics for Windows, Version 21 (IBM Corp., Armonk, NY). This approach included calculating frequencies and percentages, and the creation of charts to illustrate the distribution of DCPs across different cycles.

## Results

In cycle 1, a total of 82 DCPs were used. In body imaging, the term "likely" was most commonly used, constituting eight out of 26 phrases. Musculoskeletal imaging showed a preference for the phrase "suggestive of," appearing in five out of 13 DCPs, while neuroimaging frequently used the term "likely" in eight out of 25 phrases.

In cycle 2, there were noticeable shifts in terminology use. In body imaging, the phrase "consistent with" was notably present in three out of 10 phrases. Musculoskeletal imaging showed a predominant usage of "representing," appearing in six out of 11 DCPs. Neuroimaging favored "likely," which was used in seven out of 23 phrases.

In cycle 3, the usage of DCPs evolved further. Body imaging most commonly used "consistent with" which was seen in five out of 15 phrases. Musculoskeletal imaging frequently incorporated "representing" appearing in five out of 13 DCPs. Neuroimaging demonstrated an increased use of a definitive tone, with "likely" becoming the most common phrase, used in five out of eight phrases.

**Figure 1:**
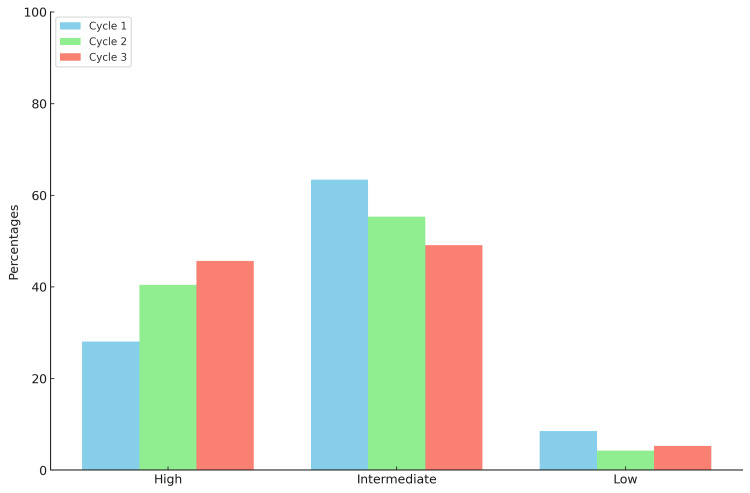
Comparative analysis of diagnostic probabilities across different cycles

The study's findings show a dynamic shift in the usage of DCPs throughout three cycles, showing a developing approach to radiological reporting. This shift in diagnostic certainty in radiological reporting is visible across the three cycles, with intermediate certainty phrases dominating initially, followed by a significant increase in high certainty phrases and a decrease in low certainty phrases, particularly in the second cycle. In terms of the overall distribution of DCPs by diagnostic probability, cycle 1 had a total of 82 DCPs, with 23 high-probability DCPs, 52 intermediate-probability DCPs, and seven low-probability DCPs. Cycle 2 had a total of 47 DCPs, with 19 high-probability DCPs, 26 intermediate-probability DCPs, and two low-probability DCPs. Finally, cycle 3 showed a mild increase to 57 total DCPs, comprising 26 high-probability DCPs, 28 intermediate-probability DCPs, and three low-probability DCPs. Figure 1 depicts the general trend of a successful shift toward more decisive and confident reporting, which was affected by focused educational interventions. It shows the percentage of high-, intermediate-, and low-probability DCPs used in each cycle.

**Figure 2:**
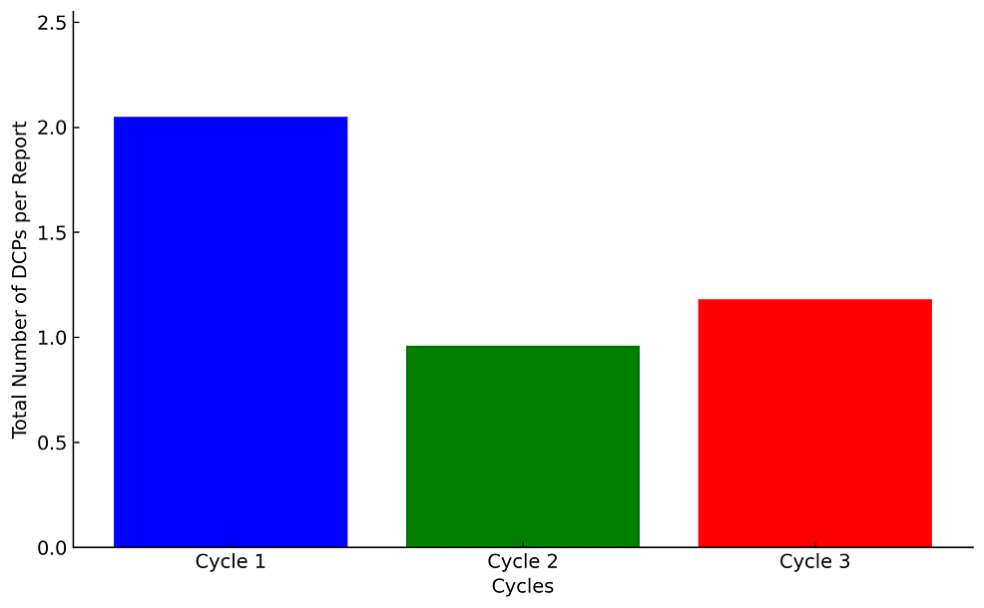
Average number of DCPs per report in each cycle DCPs, diagnostic certainty phrases

In the second cycle, there was a significant decrease in DCPs, with MRI reports containing 32 DCPs and CT reports containing 16 DCPs, both averaging approximately one DCP per report. During this cycle, there was a shift toward more decisive language in both modalities, particularly in CT reports, which frequently used phrases like "consistent with." The third cycle maintained the trend, with MRI reports containing 29 DCPs and CT reports containing 30, averaging slightly more than one per report. "Representing" was commonly used in MRI reports, whereas "likely" was more frequently used in CT reports. This trend showed a significant decrease in DCPs usage following educational intervention, followed by a minor increase, possibly reflecting a more balanced approach in diagnostic reporting (Figure 2).

## Discussion

The study found a considerable shift in the use of DCPs across three cycles of radiological reporting. The first cycle of the study was dominated by phrases that carried intermediate diagnostic certainty. After educational interventions before each of the second and third cycles, there was a gradual but noticeable shift toward the usage of phrases that carried greater diagnostic certainty and a lesser inclination toward those that carried lower diagnostic certainty. This development, which has been influenced by educational interventions, reflects a tendency toward more definitive reporting and demonstrates the importance of understanding the degree of diagnostic certainty implied by various words and phrases.

A study involving abdominal ultrasound reports in a pediatric emergency department found that uncertain reports were more likely to require additional CT scans and a longer median length of stay [15]. This higher rate of additional CT scans was likely due to the referring physicians needing additional investigations in order to devise a definitive management plan for their patients, especially when faced with the lower diagnostic certainty in the initial radiology reports. These additional investigations could have been avoided by using more certain and definitive language in the initial radiology reports. Rosenkrantz reported 75% likelihood of a follow-up imaging for “most likely a cyst, although tumor not excluded” versus 2% for the phrase "benign cyst" [16]. This increased likelihood of a follow-up imaging request is due to the usage of low DCPs. These examples reflect the negative effects of an uncertain radiology report on patient care.

Consistency in using diagnostic language in radiology reports is vital to get the message across to the referring physicians. For example, consider CT examinations of two patients with definitive findings of acute appendicitis are reported inconsistently. One is described as "findings are consistent with acute appendicitis" and the other as "findings are suggestive of acute appendicitis." This inconsistency in the usage of diagnostic terms for the same interpretation may lead to ambiguity and confusion in the minds of the referring physicians. Shinagare et al. reported inconsistency among radiologists for phrases used to express diagnostic certainty, highlighting the potential for miscommunication in radiology reports [14]. This underscores the need for interventions to improve the consistency of use of these phrases in order to enhance the quality of radiology reports, thereby improving patient care.

Given that the primary audience of radiology reports are referring physicians, it is vital that the reporting radiologist understands how words and phrases used in radiology reports are perceived by the reader. If a radiology report reads "findings are consistent with a periampullary lesion, possibly originating from the pancreatic head," it implies with high diagnostic certainty that there is a definite lesion in the periampullary area. However, the origin from the pancreatic head cannot be stated with the same level of certainty, as the use of the word “possibly” indicates a lower degree of diagnostic certainty. In this context, Khorasani et al. reported poor concordance between radiologists and non-radiologists regarding the understanding of diagnostic certainty implied by words and phrases used in radiology reports [17]. This further highlights the need for standardization of DCPs in order to get a clear message across to the audience. It also underscores the need for educational activities on radiology reporting to improve the understanding of radiologists regarding diagnostic certainty. This could lead to better communication of findings and, consequently, improved patient care.

In this perspective, the exploration of the impact of educational interventions on the use of DCPs in the current study makes an important difference in the field of radiological reporting. It highlights the relevance of continuing professional development in radiology by demonstrating a link between these interventions and greater diagnostic confidence, leading to improved patient management. In a similar quality improvement project, Unsdorfer et al. achieved commendable results in the diagnostic performance of pediatric appendiceal ultrasound [18]. This approach not only improves the accuracy of reports but also reduces the widespread issue of hedging, where overly cautious language can obscure clear decision-making. The current study indicates that targeted training can promote the use of more definitive language, thereby enabling radiologists to communicate their findings with greater confidence and clarity by minimizing excessive caution in their wording. This development is pivotal, as it helps in making more informed clinical judgments and, ultimately, better patient care. The broader implications of this research underscore the importance of ongoing education and adaptability in radiology reporting practices, especially in domains that rely heavily on precise terminology.

Limitations

This study has several limitations, including a single center, retrospective design, and a small sample size. These limitations may affect the generalizability of the results. The results, primarily reflective of a single center's reporting styles, might not accurately represent the diversity of practices in other institutions. The pathology encountered in different cycles of this study varied, along with different levels of experience of the reporting radiologists, which could further influence the diagnostic confidence. Further research with a larger and more diverse sample is necessary to corroborate our findings.

## Conclusions

The study validates the transformative impact of educational interventions on the use of DCPs in radiology reports. A significant proportion of the reports used DCPs of intermediate- to low-diagnostic certainty, underscoring the need for radiologists and trainees to understand the implications of their language choices in radiological reporting. There is an improvement of diagnostic confidence imparted in radiology reports after educational interventions. The study emphasizes the significance of continuing education to ensure the use of precise terminologies.
